# Recurrence of Human Babesiosis Caused by Reinfection

**DOI:** 10.3201/eid2710.211240

**Published:** 2021-10

**Authors:** Jonathan Ho, Erin Carey, Dennis E. Carey, Peter J. Krause

**Affiliations:** Yale School of Public Health, New Haven, Connecticut, USA (J. Ho, P.J. Krause);; University of Bridgeport, Bridgeport, Connecticut, USA (E. Carey);; Zucker School of Medicine at Hofstra/Northwell, Hempstead, New York, USA (D.E. Carey);; Yale School of Medicine, New Haven (P.J. Krause)

**Keywords:** babesiosis, human babesiosis, reinfection, recurrence, Babesia microti, parasites, piroplasm, immunity, antimicrobial drugs, tick-borne infections, vector-borne infections, United States

## Abstract

Babesiosis developed in a 62-year-old immunocompetent physician, who had an uneventful recovery after receiving atovaquone and azithromycin. Three years later, babesiosis developed again, and he was again successfully given treatment. Clinical and laboratory evidence were highly supportive of *Babesia* reinfection. Healthcare professionals should be aware that reinfection might occur in babesiosis.

## Abstract 2

Healthcare professionals must be aware that babesiosis caused by reinfection might occur.

*Babesia microti*, the primary cause of human babesiosis, is an intraerythrocytic protozoan that is transmitted by hard-bodied ticks to mammalian hosts and occasionally to humans (*1*). White-footed mice are the primary host and once infected may remain so for life. Parasitemia also persists in humans, even after antimicrobial drug therapy (*2**–**7*). Immunocompetent human hosts can experience asymptomatic infection for as long as 1 year after antimicrobial drug therapy, although most patients clear infection within several months. Patients who are immunocompromised generally have a longer duration of infection and may experience relapsing symptoms. These patients might remain parasitemic for as long as 2 years, despite antimicrobial drug therapy (*5*,*7*). Most patients recover without long-term complications, although babesiosis can result in fatal illness (*1*,*6*).

We report a case of babesiosis in a previously healthy patient who experienced a second episode of babesiosis 3 years after an initial episode. He was given a standard course of antimicrobial drugs for *Babesia* infection for each episode.

## The Study

A 62-year-old physician living in Huntington, Long Island, New York, USA, was in good health until June 9, 2013, when he felt unwell and fever, chills, headache, myalgias, fatigue, sweats, joint pain, poor appetite, and conjunctivitis developed. On the third day of illness, he noted dark urine that lasted for several days. On June 13, he was seen by his family physician, who noted fever but no other abnormality.

A complete blood count (CBC) showed a hemoglobin level of 13.9 g/dL (reference range 13 g/dL‒18 g/dL); a hematocrit of 40.8% (40%‒54%); a leukocyte count of 4,700 cells/μL (4,500 cells/μL‒11,000 cells/μL) with 55% neutrophils (54%‒62%) and 28% lymphocytes (25%‒33%); a platelet count of 68,000 cells/μL (150,000 cells/μL‒400,00 cells/μL); an aspartate aminotransferase level of 65 U/L (1 U/L‒36 U/L); and an alanine aminotransferase level of ALT 70 U/L (1 U/L‒45 U/L). He was told to return for reevaluation if symptoms did not resolve and was seen again on June 16. At that time, he had a fever (temperature 103°F). He was given doxycycline (100 mg orally every 12 h) but did not improve.

Laboratory results on June 16 showed that a *B. microti* IgM result was strongly positive (IgM titer >1:1,024, IgG titer <1:16), but *Anaplasma phagocytophilum* antibody was absent (Table). He was then given atovaquone (750 mg, 2×/d for 2 wks) and azithromycin (500 mg, 1×/d for 2 wks). He recovered completely 3 weeks after symptoms began.

**Table:** *Babesia microti* indirect fluorescent antibody test results for patient who had babesiosis 2 times, New York, USA

*B. microti* test date	Days after onset of symptoms	*B. microti* IgM titer	*B. microti* IgG titer
Episode 1, 2013 Jun 16	7	>1:1,024	<1:16
Episode 2, 2016 Jun 22	4	1:256	>1:1,024

Subsequent attempts to perform *Babesia* whole-genome sequencing on a residual blood sample obtained 3 days after the start of treatment identified *B. microti* DNA, but it was insufficient to perform full-genomic sequencing. At a follow-up visit to his physician on July 5, a CBC and tests for aspartate and alanine aminotransferase levels showed results within references ranges.

Three years later, on June 19, 2016, the patient had a fever (temperature 100°F), chills, sweats, headache, myalgias, anorexia, and difficulty concentrating develop. He also noted dark urine for several days. On June 22, he was seen by his physician, who obtained a CBC, which showed a hemoglobin level of 12.6 g/dL and hematocrit of 38.3%; a leukocyte count of 4,700 cells/μL with 55% neutrophils and 30% lymphocytes; and a platelet count of 41,000 cells/μL. A blood smear showed a *Babesia* parasitemia level of 1%. We amplified *B. microti* DNA by using PCR. Results for *B. microti* antibody were positive (IgM titer 1:256, IgG titer >1;1,024) (Table). He was then given atovaquone (750 mg every 12 h) and azithromycin (500 mg on day 1 and then 250 mg 1×/d) for 10 days, at which time symptoms had resolved. A repeat blood smear did not show any parasites. The patient has subsequently been in good health.

## Conclusions

This patient had 2 separate episodes of *B. microti* babesiosis 3 years apart. He lived in an area where *B. microti* was hyperendemic and showed typical symptoms of *Babesia* infection during each episode, including dark urine that is indicative of hemoglobinuria (*8*). In the first episode, he did not have a blood smear or PCR performed, but a high *B. microti* IgM response was suggestive of *B. microti* infection (*1**,**9*). *B. microti* infection was subsequently confirmed by identification of *B. microti* DNA. In the second episode, *B. microti* infection was confirmed by blood smear and PCR.

Both clinical and laboratory evidence support reinfection rather than relapse of infection for this patient. He was repeatedly exposed to ticks in an area where babesiosis is commonly reported (*1*). He was in good general health without evidence of immunosuppression, whereas all cases of relapsing babesiosis have been reported in immunocompromised persons. After the first episode of babesiosis, he had complete clinical recovery 2 weeks after the onset of infection and did not experience the second episode until 3 years later. In contrast, those persons who have had relapsed *B. microti* infection have all been immunocompromised, experienced relapses of infection 2 weeks to 3 months after the previous episode, and usually lack full clinical recovery between relapses (*3**–**7*). Finally, our patient had a robust *B. microti* IgM response 2 weeks after the onset of his first infection and an anamnestic antibody response with a high IgG titer on day 4 of the second infection, which is characteristic of reinfection rather than relapse. Patients who have experienced relapse have conditions that impair antibody response (e.g., B cell lymphoma, rituximab therapy, HIV/AIDS). A minimal or absent *B. microti* antibody response has been demonstrated in patients who have had relapsing babesiosis and have been tested for *B. microti* antibody (*5**,**7*).

Previous studies describe the persistence of human *B. microti* infection and clinical immunity. In a prospective study of babesiosis patients who were tested for *B. microti* DNA by PCR every 3 months after acute illness until infection cleared, parasitemia persisted up to 13 months in 22 antimicrobial drug‒treated patients and up to 27 months in 23 untreated patients (*2*). In another study, a patient was reported as having relapsing infection that persisted for 27 months (*5*). The immediate host response to *B. microti* infection is provided by innate immune elements that include the spleen, macrophages, and neutrophils. In contrast, long-term clearance of *B. microti* parasites depends in large part on antibody (*2**–**7*). Studies of the duration of *B. microti* antibody have demonstrated persistence for as little as 6 months and as long as 6 years (*2**,**6**,**10**–**11*).

Although there is strong evidence that our patient experienced reinfection, we do not have absolute confirmation, and it is possible that he could have had persistent *B. microti* infection that relapsed after 3 years. No *B. microti* PCR (or blood smear) was obtained after the initial infection. We attempted to further distinguish between relapse and reinfection by genetic sequencing of *B. microti* DNA from blood samples obtained from both episodes of infection. Unfortunately, we were unable to obtain sufficient DNA from the first episode for sequencing because a blood sample was only available 3 days after antimicrobial drug therapy was initiated, leaving few viable parasites.

In summary, our study shows evidence of reinfection after successful treatment of a *B. microti* infection. Although the evidence is highly supportive, it is not definitive. Whether our patient experienced reinfection or relapse 3 years after the initial infection, investigation of similar patients could provide useful information about the immune response to *B. microti* infection. Patients who have experienced babesiosis, and their healthcare professionals, need to be aware that babesiosis reinfection might occur, as for Lyme disease (*12*,*13*). Tickborne disease preventive measures should be maintained for patients with or without a history of babesiosis (*14*).
